# Impact of previous macrolide use on invasive pneumococcal disease due to erythromycin-resistant serotypes in adults over 59 years of age

**DOI:** 10.1007/s10096-021-04368-2

**Published:** 2021-10-31

**Authors:** Abelardo Claudio Fernández Chávez, Luis García Comas, Luis Manzano Espinosa, Jose Yuste Lobo, Octavio Corral Pazos de Provens, Jesús María Aranaz Andrés

**Affiliations:** 1 Preventive Medicine and Public Health, Hospital Universitario Ramón y Cajal, IRYCIS, Ctra. de Colmenar Viejo km. 9100, 28034 Madrid, Spain; 2 Epidemiology Service of Health Department of the Community of Madrid, Madrid, Spain; 3 Internal Medicine Service, Hospital Universitario Ramón y Cajal, Universidad de Alcalá, Madrid, Spain; 4 Pneumococcal Unit, National Centre for Microbiology, Instituto de Salud Carlos III and CIBER of Respiratory Diseases (CIBERES), Madrid, Spain; 5 Faculty of Health of UNIR, Madrid, Spain; 6 Preventive Medicine and Public Health, Hospital Universitario Ramón y Cajal, IRYCIS, CIBER of Epidemiology and Public Health (CIBERESP), Madrid, Spain

**Keywords:** Invasive pneumococcal disease, Pneumococcal conjugate vaccines, Reduced antibiotic susceptibility

## Abstract

The major goals of the study were to describe the invasive pneumococcal disease (IPD) cases due to erythromycin-resistant serotypes and to evaluate the association between these cases and recent macrolide use in individuals aged over 59 years. We selected cases of IPD reported between 2007 and 2016 in persons aged over 59 years living in the Community of Madrid (CM). We followed the European Committee on Antimicrobial Susceptibility Testing (EUCAST). The explanatory variables (age, sex, year of onset of symptoms, clinical presentation, serotypes, vaccination status) were taken from the Mandatory Notification System for Infectious Diseases System and from the Vaccination Information System. The cases were classified as either included in the 13-valent pneumococcal conjugate vaccine (PCV13) or not (nonPCV13). Associations between cases due to erythromycin-resistant serotypes and previous macrolide use (total, long and short-term) were adjusted with a logistic regression multivariate analysis. A total of 1,831 cases were identified, of whom 408 were erythromycin-resistant serotypes. PCV13 cases were associated with previous macrolide use (OR: 5.07), particularly long-acting types (OR: 8.61). NonPCV13 cases were associated with the use of total macrolides (OR: 3.48) and long-acting macrolides (OR: 4.26) suggesting that PCV13 did not reduce the IPD cases in patients with previous use of macrolides. Our results confirmed that previous macrolide consumption was associated with the presence of IPD due to erythromycin-resistant serotypes. The risk was higher with the use of long-term macrolides.

## Introduction

Invasive pneumococcal disease (IPD) due to antibiotic-resistant serotypes is a serious public health problem that can affect to population of different age groups and with high morbidity and mortality rates in individual with risk factors [1].

In addition to age, increased risk of IPD in individuals with medical conditions leading to a state of immune deficiency must be highlighted [2, 3]. However, not all the aforementioned factors are associated to a higher risk of IPD by resistant serotypes, and the relationship with previous antibiotic use is not fully understood [4–6].

Early adequate antibiotic therapy is essential for reducing the morbidity and mortality of pneumococcal infections [7]. Inappropriate use of antibiotics some days before the onset of symptoms of IPD may facilitate the involvement of resistant serotypes [6, 8]. Some studies suggest that previous macrolide consumption is associated with the resistance of *Streptococcus pneumoniae* (SP) to erythromycin, although the degree of resistance is variable [9, 10].

Practices related to inappropriate use of antibiotics include self-medication, treatment of viral diseases, and failing to complete a course of antibiotics in case of bacterial infections, among others [11, 12]. It is important to gather information about courses of antimicrobial agents used in clinical practice, their duration, and the time elapsed before the onset of the invasive infection. Such data will help us to identify cases at higher risk of being SP resistant in order to use the most appropriate empiric therapy [13, 14].

Mechanisms involved in antibiotic resistance are gene selection, competition, and transfer. Through the selection mechanism, antibiotic treatment eliminates susceptible SP, while resistant ones remain. Then, by a competitive mechanism, the resistant SP occupy the space left by the susceptible ones, increasing pharyngeal colonization [15]. Finally, the resistant SP transfer their resistant genes to the susceptible ones using processes like cannibalism [16, 17].

Some community factors are also associated with the incidence of cases of IPD due to resistant serotypes, such as childhood vaccination coverage [18, 19] and community macrolide consumption [20, 21]. Pneumococcal conjugate vaccine (PCV) reduces the pharyngeal contribution of vaccine serotypes in children under 2 years of age, preventing their transmission to adults. In the Community of Madrid (CM), the 13-valent vaccine (PCV13) has been included in the childhood vaccination schedule since 2010, except for the period between 2012 and 2015. However, it was still administered but privately financed.

The purpose of the study is to describe the invasive pneumococcal disease (IPD) cases due to erythromycin-resistant serotypes and to evaluate the association between these cases and recent macrolide use in individuals aged over 59 years.

## Materials and methods

### Selection criteria

Microbiologically confirmed IPD cases over 59 years of age residents in the CM. identified between 2007 and 2016.

Data sources

1. Mandatory Notification System for Infectious Diseases System
2. Vaccination Information System of the Community of Madrid
3. Information System and Analysis of the Pharmaceutical Service

The age of 59 years was determined, because from that age there is an adult vaccination schedule in the Community of Madrid. The calendar includes pneumococcal vaccination, since 2017.

### Variables

Age (categories: 60/69; 70/79; 80/89 and > 90) and sex.

Clinical data: date of onset of symptoms; clinical presentation (pneumonia, sepsis, bacteremia, meningitis plus sepsis and others); pneumococcal disease risk factors (chronic kidney, liver, respiratory and cardiovascular diseases, HIV, diabetes, cerebrospinal fluid fistulas, cochlear implants, anatomical or dysfunctional asplenia and patients treated with corticosteroids, immunosuppressants or immunobiological products); evolution (deceased) and data regarding hospital admission (admission to ICU and length of hospital stay).

Microbiological data: Confirmatory criteria were isolation of SP in samples from normally sterile site. The Quellung reaction was used to identify serotypes. Antibiotic susceptibility was determined by disk diffusion tests, and MIC values were obtained by the agar dilution technique in accordance the criteria established by the European Committee on Antimicrobial Susceptibility Testing (EUCAST) [22]. SP erythromycin resistance was defined as the inhibition of bacterial growth to a minimum inhibitory concentration greater than 0.5 mg/L of erythromycin.

Consumption of antibiotics during the 3 months before the onset of symptoms: total macrolide consumption, long-acting macrolides (azithromycin, telithromycin) consumption, and short-acting macrolides consumption (erythromycin, clarithromycin, spiramycin, midecamycin, josamycin) macrolides. This data could be recovered from all cases with the patient identification codes (CIPA) available.

Vaccination data: date of vaccination, type of vaccine, number of doses, and vaccination center.

Childhood vaccination coverage (CVC): We calculated the annual first vaccinated in the 2-year-old cohort, which is the age at which first vaccination with two or three doses was theoretically received under the current vaccination schedule. First vaccinated is referred to children vaccinated without the booster dose. Vaccination coverage was calculated for PCV13 (VC13). We did not consider childhood vaccination coverage with PCV10 because this vaccine was not in the childhood vaccination schedule of the CM.

Community macrolide consumption was classified by the main active ingredient (ATC) expressed as the defined daily dose (DDD) number, which is a standardized measure formulated by the WHO (Classification System with Defined Daily Doses) [23]. We calculated the defined daily dose for 1000 people (DHD), using the following formula: (DDD*1000)/(population*365).

### Analysis

We describe cases of IPD caused by resistant and sensitive strains to erythromycin. We estimated OR to compare characteristics of both groups.

The association between the presence of cases due to erythromycin-resistant serotypes and previous consumption of macrolides was analyzed using multivariate logistic models adjusting by age, sex, presence of pneumococcal disease risk factors, and childhood immunization coverage of PCV13 and community macrolide (DHD) consumption. The confidence level was 95%.

For this purpose, three logistic regression models were considered:

1. Dependent variable: total cases (0= sensitive; 1= resistant). Main variable: previous consumption of macrolides (total macrolides or short duration or long duration)
2. Dependent variable: PCV13 cases (0= sensitive; 1= resistant). Main variable: previous consumption of macrolides (total macrolides or short duration or long duration)
3. Dependent variable: non-PCV13 cases (0= sensitive; 1= resistant). Main variable: previous consumption of macrolides (total macrolides or short duration or long duration)

In the three models, the independent variables of adjustment were age, sex, presence of pneumococcal disease risk factors, childhood immunization coverage of PCV13, and community macrolide (DHD) consumption.

Confidence level was 95%.

The STATA V 14 statistical programmed was used.

## Results

**Fig. 1:**
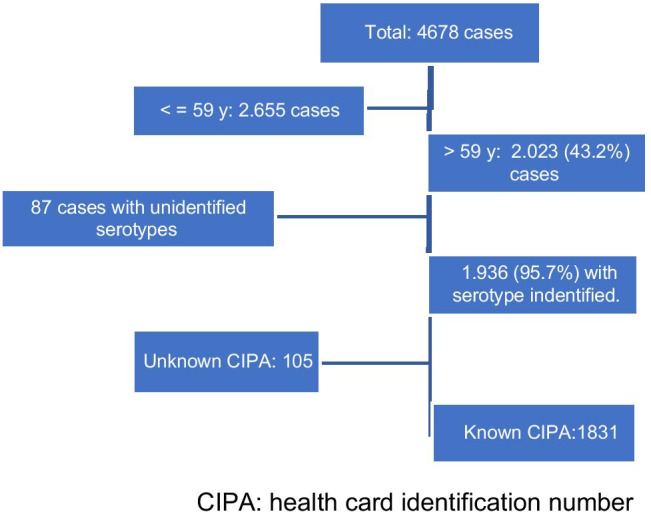
Outline of the study population size. CIPA: health card identification number

A total of 4,678 cases of IPD were reported during the study period of which 43.2% were over 59 years (2,023 cases). Serotype and antimicrobial sensitivity were identified in 95.7% of them (1936 cases), and of these the number of patients with a health card identification number (CIPA) was 1831 cases, which allowed us to obtain data on the previous use of macrolides. Finally, 425 cases with resistant strains to erythromycin were studied (21.1% of all cases over 59 years) (Fig. 1).

Among the cases caused by strains resistant to erythromycin in adults older than 59 years, 41.0% were men, 47.3% were 80 years or older, and almost 70% had at least one risk factor. The predominant clinical form was pneumonia (54.2%), followed by sepsis (16.2%). More than 85% required hospital admission (86.8%), with stays longer than 7 days for 74.2% of the admitted cases and admission to the ICU for 5.4%. The fatality rate was higher than 20%. Almost 50% had received PPV23. More than 5% had consumed macrolides in the previous 3 months (5.4%).

Cases produced by sensitive strains were similar in gender distribution and frequency of risk factors, clinical forms, and hospital admission. The prevalence of having at least one previous pathology was similar between the erythromycin-resistant serotypes and those sensitive to erythromycin. The prevalence of the clinical presentation forms was similar, with pneumonia being the most frequent. There were no significant differences between cases vaccinated with 7-valent pneumococcal conjugate vaccine (PCV7), PCV13, and polysaccharide vaccine (PPSV23) among cases with erythromycin-resistant serotypes. Regarding case evolution, this was similar in patients with erythromycin-resistant serotypes and those sensitive to erythromycin. Previous use of macrolides was higher in the cases caused by erythromycin-resistant serotypes (OR 2. 40; 95% CI 1.38–4.17) (Table 1).

**Table 1:** Characteristics of patients with IPD over 59 years of age by sensitivity to erythromycin

	Sensitivity to erythromycin				
	NSE	SE	OR_NSE/SE_	(CI 95%)	
Sex	%	%	OR	Lower	Upper
Men	51.0	56.9	0.79	0.63	0.98
Age	%	%	OR	Lower	Upper
60–69	27.0	28.0	0.95	0.74	1.21
70–79	25.7	32.4	0.72	0.56	0.93
80–89	35.5	30.5	1.26	0.99	1.58
> 90	11.8	9.1	1.34	0.94	1.90
Pneumococcal disease risk factors	%	%	OR	Lower	Upper
One or more	68.9	68.6	1.02	0.88	1.19
Clinical presentation	%	%	OR	Lower	Upper
Pneumonia	54.2	57.5	0.87	0.70	1.09
Sepsis	16.2	17.7	0.90	0.67	1.21
Bacteremia	15.9	13.6	1.21	0.89	1.64
Meningitis y sepsis	7.4	7.6	0.97	0.64	1.47
Others	6.4	3.7	1.80	1.11	2.90
Evolution	%	%	OR	Lower	Upper
Hospitalized	86.8	89.2	0.80	0.57	1.11
Hospital stay longer than 7 days	74.2	72.6	1.08	0.79	1.47
Admission to ICU	5.4	8.2	0.64	0.40	1.02
Deaths	20.8	19.3	1.10	0.84	1.45
Vaccination status	%	%	OR	Lower	Upper
Vaccinated with PCV7	0.2	0.4	0.58	0.07	4.83
Vaccinated with PCV13	1.2	1.8	0.69	0.26	1.82
Vaccinated with PPSV23	48.8	48.9	0.99	0.80	1.24
Macrolide’s consumption (previous 3 months)	%	%	OR	Lower	Upper
Macrolides (total)	5.4	2.3	2,40	1,38	4,17
Long-duration macrolides	3.4	1.3	2,77	1,37	5,63
Short-duration macrolides	2.0	1.1	1,75	0,75	4,14
TOTAL (*N*)	408	1423			

*NSE* non-sensitive serotypes to erythromycin, *SE* sensitive serotypes to erythromycin

Multivariate logistic regression models showed an association between erythromycin-resistant serotype cases and previous use of long-acting macrolides (OR: 5.18 (95% CI: 2.30–11.66)), adjusted by the other individual (age, gender, and IPD risk factors) and population factors (childhood vaccination coverage of PCV13 and community macrolide consumption). Previous use of short-term macrolides was not related to a higher risk of erythromycin-resistant serotype involvement.

The association between PCV13 erythromycin-resistant serotype cases and previous macrolide consumption was greater (OR: 5.07 (95% CI: 1.64–15.64)) than between non-PCV13 cases (OR: 3.48 (95% CI: 1.58–7.69)) (Table 2).

**Table 2:** Association between erythromycin-resistant serotype IPD cases and previous macrolide consumption. Multivariate logistic regression

	Model 1. Total			Model 2. PCV13			Model 3. Non-PCV13		
	OR	CI95%		OR	CI95%		OR	CI95%	
Previous macrolides use (total macrolides)	3.83*	2.03	7.24	5.07*	1.64	15.64	3.48*	1.58	7.69
PCV13 (childhood vaccination coverage)	0.90	0.72	1.12	0.94	0.66	1.34	0.93	0.69	1.24
DHD (community macrolide consumption)	0.58	0.14	2.43	3.16	0.26	37.75	0.28	0.05	1.72
IPD risk factors (one or more)	1.09	0.77	1.54	0.97	0.56	1.68	1.22	0.77	1.93
Gender	0.81	0.60	1.1	0.74	0.44	1.26	0.83	0.57	1.23
70 a 79 y	0.99	0.65	1.52	0.72	0.35	1.51	1.16	0.69	1.97
80 to 89 y	1.35	0.92	1.99	1.24	0.65	2.37	1.41	0.86	2.31
≥ 90 y	1.49	0.88	2.53	1.53	0.66	3.54	1.37	0.68	2.75
Previous macrolides use (long duration macrolides)	5.18*	2.30	11.659	8.61*	1.99	37.20	4.26*	1.54	11.77
PCV13 (childhood vaccination coverage)	0.89	0.71	1.11	0.91	0.64	1.30	0.93	0.69	1.24
DHD (community macrolide consumption)	0.58	0.14	2.43	3.47	0.29	41.62	0.28	0.05	1.68
IPD risk factors (one or more)	1.10	0.78	1.56	0.99	0.57	1.72	1.22	0.77	1.93
Gender	0.80	0.59	1.09	0.72	0.42	1.21	0.84	0.57	1.23
70 to 79 y	0.96	0.63	1.47	0.68	0.32	1.42	1.14	0.67	1.92
80 to 89 y	1.32	0.90	1.95	1.20	0.63	2.27	1.39	0.85	2.27
≥90 y	1.48	0.87	2.50	1.44	0.63	3.32	1.38	0.69	2.78
Previous macrolides use (short duration macrolides)	2.06	0.76	5.59	1.57	0.27	9.03	2.43	0.72	8.29
PCV13 (childhood vaccination coverage)	0.36	0.72	1.12	0.95	0.67	1.34	0.93	0.70	1.24
DHD (community macrolide consumption)	0.65	0.16	2.73	3.49	0.30	40.42	0.32	0.05	1.93
IPD risk factors (one or more)	1.08	0.77	1.53	0.96	0.56	1.65	1.21	0.77	1.91
Gender	0.80	0.59	1.09	0.74	0.44	1.24	0.83	0.57	1.22
70 to 79 y	0.97	0.64	1.48	0.74	0.36	1.52	1.13	0.67	1.91
80 to 89 y	1.26	0.86	1.85	1.12	0.60	2.11	1.34	0.82	2.19
≥ 90 y	1.42	0.85	2.39	1.41	0.62	3.22	1.31	0.66	2.63

*PCV13* 13-valent pneumococcal conjugate vaccine, *DHD* daily defined dose per 1,000 persons per day, *IPD* infection pneumococcal disease

*p < 0.05

## Discussion

According to our analysis, previous use of macrolides increases the risk of cases of IPD due to erythromycin-resistant serotypes. These results are consistent with some other studies, such as a prospective Canadian surveillance study, which concluded that exposure to a class of antibiotics is the most important factor in predicting antimicrobial resistance of SP [24]. The period prior in our study was 3 months, enough time for changes in pharyngeal colonization to occur.

There were differences in the risk of IPD due to resistant serotypes according to the different types of macrolides previously consumed. We detected a higher risk with long-term macrolides. This finding has been observed in other published studies [25, 26], such as that carried out by Keenan, which observed an increase in resistant cases after treatment with azithromycin [27, 28]. According to our study, the risk was the same for cases of vaccine and non-vaccine serotypes.

Published studies indicate that most antibiotics are prescribed in the primary care setting [29]. For our analyses, data on previous macrolide consumption were obtained from the outpatient setting. The greater use of antibiotics in Spain can be attributed to the “pro-antibiotic” culture, present in the population of southern Europe [30], supported by the “preventive care” approach by the primary care physician of prescribing antibiotics even in the case of low risk of bacterial infection, such as upper respiratory infections, which in most cases are viral. The Spanish Medicines Agency launched a strategic and action plan in 2014 to reduce the risk of resistance to antibiotics [31]. The Plan has established among its strategic lines: to monitor the consumption and resistance to antibiotics; control bacterial resistance; identify and promote alternative and/or complementary prevention and treatment measures; training and information for health professionals; and communication and awareness of the population as a whole and of population subgroups. Among the action proposals aimed at patients are promoting educational campaigns aimed at the population to facilitate the proper use of antibiotics and ensuring that medical prescriptions are necessary for their dispensing.

In our study, the risk of cases of IPD due to resistant serotypes was adjusted for age, sex, history of disease, community use of antibiotics, and routine childhood vaccination. According to the scientific literature and our multivariate models, these adjustment variables were most likely to influence the occurrence of cases due to resistant serotypes. These factors had different effects, such as the link between previous macrolide consumption and routine childhood vaccination.

Age was one of the most significant individual factors. Bivariate analyses show that there is an increased risk of IPD due to resistant serotypes in older persons. This could be due to the effect of immunosenescence on the immune response to infectious diseases, which entails greater antibiotic consumption, with the consequent increase in resistance [32]. According to our analyses, cases over 70 years of age are at higher risk of developing invasive diseases. The relationship between gender, development of IPD, and antibiotic resistance was also analyzed. Our results showed that men had an increased susceptibility to IPD. However, this risk seemed not to be associated with the development of IPD cases caused by resistant serotypes.

Pathological history and previous vaccination were also analyzed in the present study [5, 6, 33, 34]. We did not find the presence of a pathological history as a risk factor for IPD due to resistant serotypes. It is believed that immunosuppression and chronic respiratory disease predispose to antibiotic resistance [35], since both conditions imply higher antibiotic use and, therefore, greater likelihood of antibacterial resistance. Unfortunately, we did not have categorized data of the pathological history for our study.

Previous vaccination with PCV was not considered in multivariate analyses because of the low coverage among adults during the study period. During this period, the vaccine was recommended for patients with a history of disease until 2017. It was included in the adult calendar for persons older than 59. Concerning PPV23, the percentage of vaccines was higher among cases of resistant serotypes, a situation associated with the vaccination criteria involving the population over 59 years of age and with a pathological history. We did not consider the effect of the coverage of PPSV23, partly because the scientific literature does not give it a significant role in the epidemiology of resistant serotypes [30]. Unlike conjugate vaccines, PPSV23 does not generate lymphocyte-related immune memory nor does it intervene in pharyngeal colonization of SP, both important processes that explain the transmission and dissemination of antibiotic-resistant serotypes

Among the different factors analyzed in our population study, it is important to highlight that systematic pediatric vaccination had an indirect effect in adults, reducing IPD cases caused by serotypes associated to antibiotic resistance [18, 36–38], and even with macrolide consumption in the community. We did not find a direct correlation between vaccine coverage and macrolide consumption, being indeed opposite to it was expected. The indirect effect of childhood vaccine coverage (PCV13), of children under 2 years, who are the main reservoirs and transmitters of SP to the rest of the population, has had an important role in the reduction of the resistant serotype-based IPD in people over 59 years of age [18].

On the other hand, the increase of IPD due to resistant serotypes may imply a worsening of the evolution of the IPD, because of the narrower therapeutic arsenal [39, 40]. However, we have not detected higher mortality or incidence of more severe clinical forms or worse clinical evolution of cases due to resistant serotypes, as reported in other studies [33, 41].

The main strength of this study is the quality of the individual data and the powerful data of the Mandatory Epidemiological Surveillance Network of CM. One of the weaknesses of the study is a lack of data to allow us to know if the changes identified are due to other factors such as the secular trend in the disease, changes in the notification, or random changes [42]. Neither did we consider other intervening factors such as socioeconomic factors or sub-categorization of pathological history.

We can conclude that there is a greater risk of IPD due to resistant serotypes after being treated with long-duration macrolides, considering the individual and population factors involved. This work supports the new policies of the Spanish health authorities, especially those related to the promotion of alternative and complementary measures for the prevention and treatment of infectious diseases of the upper tract, in order to avoid the selection of multidrug resistant strains and the emergence of non-vaccine serotypes associated to antibiotic resistance.

## Data Availability

Yes. The datasets generated during and/or analyzed during the current study are available from the corresponding author on reasonable request.

## Code availability

Yes.
